# Characterization of the Spatial Distribution of the Pepper Weevil, *Anthonomus eugenii* Cano (Col.: Curculionidae), in Pepper Fields in South Florida

**DOI:** 10.3390/insects15080579

**Published:** 2024-07-30

**Authors:** Victoria O. Adeleye, Dakshina R. Seal, Xavier Martini, Geoffrey Meru, Oscar E. Liburd

**Affiliations:** 1 Tropical Research and Education Center, Entomology and Nematology Department, University of Florida’s Institute of Food and Agricultural Sciences (UF/IFAS), Homestead, FL 33031, USA; 2 North Florida Research and Education Center, Entomology and Nematology, University of Florida, Quincy, FL 32351, USA; xmartini@ufl.edu; 3 Tropical Research and Education Center, Horticultural Sciences Department, University of Florida, Homestead, FL 33031, USA; gmeru@ufl.edu; 4 Horticultural Sciences Department, University of Florida, Gainesville, FL 32611, USA; 5 Entomology and Nematology Department, University of Florida, Gainesville, FL 32611, USA; oeliburd@ufl.edu

**Keywords:** spatial distribution, aggregated, Geary’s C, Moran’s I, SADIE

## Abstract

**Simple Summary:**

Pepper weevil (*Anthonomus eugenii* Cano, 1894) poses a significant threat to pepper crops worldwide in tropical and sub-tropical regions. Despite its importance, the spatial distribution of pepper weevil in fields using geospatial methods like SADIE, Moran’s I, and Geary’s C remains unexplored. Few studies have utilized mean-variance-based methods to assess pepper weevil distribution. This study aimed to elucidate the distribution of pepper weevil in seven pepper fields located in South Florida, Miami Dade County, while also discerning any differences across fields or similarities in distribution patterns. The results revealed an aggregated distribution of pepper weevil, which tended to become more regular towards the end of the season as fruits and plants matured. Importantly, this aggregated distribution pattern observed through the geospatial techniques aligns with findings from previous studies utilizing mean-variance-based methods. Additionally, our study highlights that infested fruit counts can serve as an effective indicator of pepper weevil distribution, reducing the time required for adult population assessment. Given the cryptic nature and small size of pepper weevil adults, visual detection on plants can be challenging, whereas infested fruits are readily observable.

**Abstract:**

The pepper weevil, *Anthonomus eugenii* Cano, is an economically important pest of cultivated peppers (*Capsicum annuum*) in tropical and subtropical regions of the world. This study aimed to ascertain the spatial distribution of pepper weevil infestation across various fields in Miami Dade County, South Florida. The spatio-temporal dynamics of pepper weevil were evaluated using 144 sample points within each of seven pepper fields. The data were analyzed using three different geospatial techniques, spatial analysis by distance indices (SADIE), Moran’s I, and Geary’s C, to determine the spatial distribution of pepper weevil. The SADIE analysis revealed a significant aggregation distribution in 18 out of 30 sampling dates across all fields. The results from Geary’s C and Moran’s I indices indicated a positive spatial autocorrelation (spatial clustering/aggregation) of pepper weevil regardless of field or pepper types. Overall, the findings from this study depict an aggregated spatial distribution pattern of pepper weevil populations, characterized by a tendency for aggregation that transitions to a more uniform distribution as the season progresses.

## 1. Introduction

Pepper weevil, (*Anthonomus eugenii* Cano) is a destructive pest in almost all cultivated peppers [1]. Once the field is infested, it is difficult to manage because the immature stages develop within the fruits or other plant parts. Thus, products applied for the management of pepper weevils only target the adult stage [1,2]. Pepper weevil males produce an aggregation pheromone that has been synthesized and sold commercially [3,4]. However, field studies are needed to understand pepper weevil spatial distributions in fields and across seasons.

The management of pepper weevil is a serious concern to pepper growers. Growers manage this pest primarily with the repeated use of insecticides. However, only a few insecticides, including Thiamethoxam (Actara) and Oxamyl (Vydate) are available for pepper weevil suppression in South Florida. This is because pepper weevil has developed resistance to many of the available insecticides. Sampling techniques used for pepper weevil include inspecting terminal buds [5,6]; using pheromone-baited yellow sticky traps [7]; sequential sampling where the first plant is determined randomly, then every tenth plant after the first is sampled [8]; and sampling pepper weevil damage rather than pepper weevil adults [9]. However, for management decisions, direct adult counts are the most accurate for determining whether management is needed. Whole plants were inspected for adult weevil counts and found to be less precise than the terminal bud count [10]. In bell pepper fields, the counts of pepper weevil observed on visible terminal buds at the apex of the plant canopy were notably higher in the morning compared to those in the afternoon, with variations apparent between blocks and quadrat sampling sizes. Consequently, to accurately determine hotspots, a random sampling method was recommended rather than the systematic sampling used in the study [10].

The main issue with the currently available research is that immature life stages are not considered because they are not easily accessed. This is significant as it is the immature stage that significantly damages the fruit and makes it unmarketable. Furthermore, adult pepper weevils can be difficult to spot during scouting or sampling. It has also been reported that the adult pepper weevil distribution pattern is clumped [6,8], making it difficult to locate through regular scouting using the visual method. This may cause underestimation (sampling random abundance spots) or overestimation (sampling hot spots) of absolute population abundance. Therefore, for many growers, fruit drops with yellow calyx are signs of infestation in pepper fields [11]; however, pepper weevils at this stage are already established and insecticide treatments are most likely inefficient (based on personal observations).

Three common distribution patterns followed by insects include the regular (binomial, i.e., when a pest is distributed evenly within the sampling universe), random (Poisson, i.e., when a pest is haphazardly distributed across the sampling universe), and clumped (negative binomial, i.e., the pest exhibits aggregated patterns across the sampling universe) distributions [12]. Understanding the distribution pattern of important agricultural pests, as well as environmental and biological factors that influence their distribution and abundance, is vital for the successful development of an effective management program and using tools in a cost-effective manner. In addition, a reliable sampling method can be developed knowing the actual distribution and dispersion patterns within fields and within plants [13].

Factors such as previous crop history, habitat, and population age influence pest abundance and distribution in fields. For instance, wireworms were reported to show an aggregated spatial distribution in sweet potato, *Ipomoea batatas* L., fields in Georgia at the beginning of the season. However, they showed a random distribution during the latter part of the season [14]. The distribution pattern of melon thrips, *Thrips palmi* Karny, common blossom thrips, *Frankliniella schultzei* Trybom, western flower thrips, *Frankliniella occidentalis* Pergande, and tomato chlorotic spot virus (TCSV) transmitted by thrips, during a three-year study in three commercial tomato fields in South Florida was mostly regular and aggregated [13]. The alfalfa weevil, *Hypera postica*, was reported to follow a negative binomial distribution pattern, which signifies a clumped or aggregated distribution [14]. The boll weevil, *Anthonomus grandis* Boheman, squares with eggs, the adult feeding damage to squares, and the adults mostly fit a negative binomial distribution [15]. All the above-mentioned insects show an aggregated distribution pattern in most cases, especially the boll weevil, which is the same genus and similar in size to the pepper weevil. However, their lifestyles are different.

Multiple indices are used when studying the spatial distribution of an insect [16,17,18]. Indices like Taylor’s power law and Iwao’s method use mean-variance-based methods and are mainly focused on the frequency distribution of counts and not their location [19], while other indices including Variogram and SADIE use spatial methods. Spatial autocorrelation statistics measure the degree of dependency in a dataset taken from a geographic location [20]. Other indices include the index of dispersion (VMR), Green’s index or Green’s coefficient (C), mean crowding (mx), Lloyd’s index of patchiness, or Lloyd’s mean crowding (m*).

All the indices mentioned above are valuable tools for understanding the spatial patterns of insect distribution. They help researchers make informed decisions regarding insect management, conservation efforts, and ecological studies. They also provide a quantitative framework to assess spatial variability and autocorrelation in insect populations across different scales. In this study, the authors have used several indices to assess the distribution of the pepper weevil on jalapeño and long hot pepper. The information derived from this study will be used to construct an important avenue to develop a sustainable management program against the pepper weevil and improve sampling techniques and insecticide timing.

This study aimed to determine within the field the spatial distribution of pepper weevil damage by sampling pepper-weevil-infested fruits rather than pepper weevils in the adult stage. For this reason, the sampling parameter used was the number of pepper-weevil-infested fruits.

## 2. Materials and Methods

### 2.1. Within Field Distribution of Pepper Weevil on Long Hot and Jalapeño Peppers

The distribution of pepper weevils was studied in Homestead, Florida, in seven fields on widely grown long hot (4) and jalapeño peppers (3). Four fields were used during the 2020 season, two fields during the 2022 vegetable season, and one field in 2023 (Table 1). Each pepper cultivar was transplanted on raised beds, 91.4 cm wide and 10.2 cm high, in 0.10 ha. Crops were managed (irrigation, fertilization, weed removal, and fungicide application) using standard production practices as described in the vegetable handbook of Florida [21]. Each field was divided into 48 sampling points (0.002 ha). Three plants in each sampling point (3 plants × 6 columns × 8 rows = 144 plants) were randomly sampled for pepper-weevil-infested fruits. All infested fruits were collected from each of the plants sampled. Sampling began nine weeks after planting (the last week in December in the first field, the second and third weeks in January in the second field, and the first week in January in the third field). In each field, samples were conducted at least 4 times at weekly intervals.

### 2.2. Collection and Fruit Dissection

Infested fruits (fruit with a yellow calyx either still hanging on the plant or had dropped prematurely) collected from each section in each field were placed in a 473 mL or 946 mL plastic container with a lid (Uline Deli, Naples, FL, USA) and marked with the field number, section, and cultivar. All fruits were then transported to the Vegetable IPM Laboratory in Homestead, where infested fruits were dissected for confirming pepper weevil infestation. The number of pepper-weevil-infested fruit was counted and recorded for each sampling point. The sampling was conducted four times at weekly intervals (during date to date).

Field 1 of 2020 (25.62°, −80.47°): The raised beds covered with white-on-black plastic mulch ran from south to north. The field was planted with jalapeño peppers. The west and south ends were bordered by a street, the north end by an ornamental farm, and the east end by a mixed vegetable field. The field was planted in the last week of October 2020.

Field 2 of 2020 (25.43°, −80.47°): The raised beds covered with white-on-black plastic mulch ran from south to north. The field was planted with long hot pepper cultivar. The north and the east ends were bordered by a farm, the south end was bordered by a vegetable farm, and on the west side was a street. The field was planted in the last week of October 2020.

Field 3 of 2020 (25.58°, −80.52°): The beds ran from east to west. The field was bordered by a residential house to the south and a horse farm to the east. The north side of the field was bordered by a road and the west side by a residential house. The field had jalapeño pepper cultivar. The field was planted in the first week of November 2020.

Field 4 of 2020 (25.58°, −80.52°): The beds ran from east to west. The field was bordered by a residential house to the west and a horse farm to the east. The north side of the field was bordered by a road and the south side by a vegetable field. The field had long hot pepper cultivar. The field was planted in the first week of November 2020.

Field 5 of 2022 (25.62°, −80.49°): The raised beds covered with white-on-black plastic mulch ran from south to north. The field was planted with jalapeño pepper cultivar (4000–8000 Scoville units). The west and south end parts of the field were bordered by a street, and the east end parts were bordered by an ornamental and fruit tree farm. On the north side was a street that led to a major road. The field was planted in the last week of October 2021.

Field 6 of 2022 (25.62°, −80.49°): The raised beds covered with white-on-black plastic mulch ran from south to north. The field was planted with long hot pepper cultivar (100–1000 Scoville units). The west end part of the field was bordered by a street and the south end part was bordered by a residential house. The east side was bordered by a vegetable farm and on the north side was a street that led to a major road. The field was planted in the last week of October 2021.

Field 7 of 2023 (25.43°, −80.52°): The raised beds covered with white-on-black plastic mulch ran from south to north. The field was planted with long hot pepper cultivar. The north end part of the field was bordered by a road and the south end part was bordered by a farm. The east side was a road, and the west side was a farm with residential housing. The field was planted in the last week of October 2022.

In all fields, each study area consisted of 22 raised beds, each 36.6 m (120 ft) long, 91.5 cm wide, and 10.2 cm and 3 cm in center-to-center spacing between two adjacent beds. Each bed was divided into six 6.1 m long plots. The raised beds were covered with white-on-black polyethylene mulch (Canslit Inc., Victoriaville, QC, Canada, and supplied by IMAFLEX USA Inc., Thomasville, NC, USA). Two drip tubes (RO-Drip, Rivulis Irrigation Inc., San Diego, CA, USA) with emitting holes spaced 30 cm apart were placed on the beds for irrigation. In each location, pepper transplants were set in two parallel rows per bed with 30 cm spacing between plants and 60 cm between rows.

### 2.3. Statistics Used to Determine Distribution Patterns

The data on the pepper weevil field distribution pattern were subjected to various statistical indices (Table 2).

### 2.4. Moran’s I and Geary’s C

Moran’s I is a measure that determines whether spatial patterns in a given population are clustered, dispersed, or random [22]. This index quantifies the degree of similarity or dissimilarity between values at different locations, aiding researchers in understanding the spatial structure of an insect population. Moran’s I detects spatial dependence in a dataset [23]. Moran’s I ranges from −1 to 1. Positive values indicate positive spatial autocorrelation (spatial clustering), negative values indicate negative spatial autocorrelation, and a value of zero represents no spatial autocorrelation [24]. Z and *p* values signify whether differences are statistically significant or not. A positive z value with a statistically significant *p* value shows that similar values cluster spatially (aggregation), while a negative z value that is statistically significant shows that similar values are dispersed spatially (regular), and when the *p* value is not statistically significant, it is possible that there is complete spatial randomness (random distribution) [24].

Geary’s C index is calculated by comparing the weighted average of squared differences between values at different locations to the global variance of the dataset [22,24]. Geary’s C values range from 0 to 2. A zero value indicates a positive autocorrelation or a spatially clustered pattern (aggregation), while a value of 2 indicates a negative autocorrelation or spatial dispersion (regular). A value near 1 indicates a negative autocorrelation or a random spatial pattern (random) [24,25,26]. According to [27], when the mean equals 1, it indicates no spatial autocorrelation; when the *p* value is significant and the values are between 0 and 1, it indicates a positive spatial autocorrelation, while a negative spatial autocorrelation would have values greater than 1 with a significant *p* value.

### 2.5. SADIE Index

The SADIE (spatial analysis by distance indices) quantifies the spatial distribution or arrangement of insects or features within a given study area. Compared with traditional approaches, SADIE uses all the information in the sample within a given space [28,29]. Data on the location or the distribution of insects are collected from a study area. The boundaries of the study area are clearly defined. For count data, SADIE requires the two-dimensional coordinates of each sample unit and the associated count. However, it places no restrictions on how the sample units are arranged [28]. The results of a SADIE analysis can provide insights into the spatial pattern of the subject of interest being studied (in this case, pepper weevil). The overall index of aggregation is used to determine spatial patterns. If the observed indices equal one, it indicates a random spatial distribution. If the index is greater than one, it indicates a clustered or aggregated spatial pattern. If the observed index is less than one, it indicates a uniform or regular spatial pattern [19,28,30,31,32,33]. The percentage probability (Pa value) of the index of aggregation (Ia) determines the statistical significance of aggregation [34,35].

Taylor’s power law and Iwao’s patchiness regression are mainly based on mean–variance relationships, but they focus on frequency distribution of counts and not their location [19]. An insect population follows a random distribution pattern when the slope (*b* value) is not significantly different from 1, an aggregation distribution when the slope is significantly > 1, and a uniform or regular distribution when the slope is significantly < 1.0 (*p* < 0.05).

### 2.6. Data Analysis

For the spatial analysis using SADIE, the devtools package [36] was first installed, then the epiphy package [37] from github was installed (R Software, version 4.3.1). The total fruit count of pepper-weevil-infested fruit by date and field was subjected to processes through SADIE to compute the index of aggregation.

To investigate spatial autocorrelation in the dataset, the SAS software package was utilized employing the Proc Variogram procedure. The analysis was conducted using two different weighting schemes: distance-based and binary-based weights. The total number of infested fruit (count data) was the variable of interest for which spatial autocorrelation was computed. Geary’s C and the Moran’s I coefficients and the Z and *p* values were obtained and reported in tables (SAS package 9.4) [38].

## 3. Results

### 3.1. Moran’s I and Geary’s C Indices

Moran’s I index proved positive spatial autocorrelation with statistically significant ‘*p*’ values on all sampling dates on long hot pepper in Fields 2, 6, and 7 (Table 3). The ‘z’ values were positive and statistically significant, indicating that similar values were spatially clustered. The same trend was observed on the jalapeño pepper in Field 1 and the long hot pepper in Field 4, except for the last sampling date where the ‘*p*’ value was not significant (Table 3). *p* values were not statistically significant on the first (9 WAP), third (11 WAP), or fourth (12 WAP) sampling dates on the jalapeño pepper in Field 3. However, Moran’s I index was positive, indicating positive spatial autocorrelation. The *p* value was statistically significant with a positive z value, which indicated that similar values clustered spatially on the second sampling date (10 WAP) for the jalapeño pepper in Field 3 (Table 3).

For the jalapeño pepper in Field 5, the Moran’s I values were negative with insignificant *p* values on the first and last sampling dates, which indicated a weak negative spatial autocorrelation. However, on the second (10 WAP), third (11 WAP), and fourth (12 WAP) sampling dates, the *p* values were statistically significant with positive Moran’s I indices, which indicated a positive autocorrelation (Table 3).

In Field 2 with the long hot peppers, the Geary’s C values indicated a clustered spatial pattern and a positive spatial autocorrelation. In Field 1 with the jalapeño peppers, the *p* values were significant on the second (10 WAP) and third (11 WAP) sampling dates and had values between 0 and 1, which indicated a positive spatial autocorrelation. However, there was no spatial autocorrelation on the first sampling date. In Field 4 with the long hot and Field 3 with the jalapeño peppers, the *p* values were significant on the first (9 WAP) and second (10 WAP) sampling dates. They had Geary’s C values between 0 and 1, which indicated a positive spatial autocorrelation. However, there was no spatial autocorrelation on the third sampling date. The significant values between 0 and 1 in the long hot pepper Fields 6 and 7 were indicators of a positive spatial autocorrelation on all sampling dates. However, the *p* value was not significant on the last sampling date (13 WAP) in the long hot pepper Field 6 (Table 3). In summary, 21 out of 28 sampling dates and 18 out of 28 sampling dates using Moran’s I and Geary’s C indices, respectively, showed positive spatial autocorrelation in most of the fields on the first sampling dates, the later dates showing a regular or a random distribution pattern. However, there was an exception in one of the jalapeño fields that fluctuated in its distribution pattern for both Moran’s and Geary’s indices (Jalapeño Field 5).

### 3.2. SADIE Index

The aggregation was statistically significant in three out of five (60%) sampling dates, and one out of four (25%) sampling dates in jalapeño and long hot fields, respectively, in the first year (Table 4). In the second year, the results indicated that aggregation was statistically significant in one out of four (25%), and in two out of four (50%) sampling dates in the jalapeño and long hot fields, respectively. The aggregation was statistically significant in two out of six (33.3%) sampling dates, and four out of five (80%) sampling dates in jalapeño and long hot fields, respectively, in the third year (Table 4).

In the ‘long hot’ pepper field in year 2, on the first sampling date (9 WAP), the aggregation was statistically significant (*p* = 0.04). On the second sampling date (10 WAP), the index of aggregation (Ia) was greater than 1 (1.3), which showed an aggregated distribution. However, the *p* value for the aggregation index was not statistically significant (*p* = 0.08). The counts on the third and fourth sampling dates, (11 WAP and 12 WAP), respectively, showed a regular and random distribution pattern, respectively. In the jalapeño field in year 1, the indices of aggregation (Ia) were significant on the first (9 WAP), second (10 WAP), and third (11 WAP) sampling dates. The distribution pattern became random (Ia = 1) on the fourth (12 WAP) and regular (Ia < 1) on the fifth (13 WAP) sampling dates (Table 4).

According to the SADIE results, in the long hot Field 4, the indices of aggregation on the first and second sampling dates (9 WAP and 10 WAP) indicated a significantly aggregated pattern and became random at later dates. In the jalapeño Field 3 in year 1, the aggregation index was statistically significant on the first sampling date (9 WAP) but later became insignificant at later dates (Table 4). In the long hot Field 6 in year 2, the indices of aggregation indicated a statistically significant aggregation (*p* = 0.02, *p* < 2.22 × 10^−16^) except for the fourth sampling date (12 WAP) (*p* = 0.07). In the jalapeño Field 5 in year 2, there was a fluctuation between aggregated and random distribution patterns (Table 4). In Field 7, on the only sample date in the long hot pepper field in the third year, the aggregation index (Ia) was statistically significant, and the counts showed an aggregation distribution pattern (Table 4). In summary, using SADIE, the aggregated distribution pattern for pepper-weevil-infested fruit was statistically significant (*p* < 0.05) in 14 out of 29 (48.3%) sampling dates in all fields, 8 out of 14 (57%) in long hot fields, and 6 out of 15 (40%) in jalapeño fields (Table 4). Overall, the pepper weevil distribution was aggregated on the first and second sampling dates, and the distribution became either random or regular at later dates. However, the long hot Field 6 in year 2 showed an aggregated spatial distribution on all sampling dates (Table 4).

## 4. Discussion

This was the first study to report the spatial distribution of pepper weevil using Moran’s I and Geary’s C indices, and SADIE. There was more spatial aggregation of the pepper weevil in the early growing dates of pepper. The distribution tended to become random or regular at later dates with the vegetative growth of plants and the production of more flowers and fruits. This was evident in the results obtained using SADIE (Table 4): they were aggregated in the first weeks of sampling and later became regular or random at later dates. Moran’s I and Geary’s C indices showed similar trends compared to the SADIE index. This pattern could be explained by the fact that pepper weevil males produce aggregation pheromones [39,40,41], which could be a possible reason for the aggregated distribution. Later in the season, all plants in the study area produced flowers and fruits providing more resource habitat; therefore, pepper weevil adults could easily be found on almost every plant observed (personal observation).

Pepper weevil aggregation was earlier reported [8] using mean-variance-based methods. The results from this study further supported the previous findings. The negative binomial distribution predicted the adult pepper weevil distribution in the study where adults were counted directly on plants using sequential sampling [8], while in the case of this present study, the number of infested fruits was counted. A study reported that the number of adults found on different plant parts and during different times varied; therefore, infested fruits could better explain the amount of damage caused by pepper weevil adults in pepper fields. Pepper weevil adult dispersal using terminal bud inspection was aggregated [6,10].

At the initial phase of infestation, pepper weevil adults may follow some general or host-specific cues, which include visual cues, olfactory cues, aggregation pheromone produced by males, herbivore-induced volatiles, and/or constitutive host plant volatiles [42]. The level of these cues varies in different locations in a field. It could cause variation in the distribution pattern because feeding damage by herbivores can change the quantity and quality of the volatiles emitted [3]. Host plant volatiles play a crucial role in insects’ chemical communication, dispersal, and host-finding behavior, such as those of pepper weevil [41,42,43]. Pepper weevils can orientate towards volatile organic compounds (VOCs) emitted by plants with or without visual or pheromone cues. So, it is possible that while other herbivores or pathogens attack pepper plants, they emit these VOCs, which then influence the plant’s initial arrival and pepper weevil infestation, and this can start at any part of the field, especially the field edges [42]. After their arrival to the field, specific contact or short-range cues can influence the acceptability or rejection of the plant for oviposition and survival. The subsequent arrival of more weevils to the field may be influenced by either the aggregation pheromone cues or the combined effect of both plant volatiles and aggregation pheromone, if the first weevil that arrived was a male. If the first weevil is a female, her feeding or oviposition will induce volatiles that will assist other weevils in the host plant location [42].

Previous studies have reported the presence of oviposition-deterring and host-marking pheromones in oviposition plugs and female frass of pepper weevil [3,44]. This could be another reason why females avoid ovipositing on already-infested fruits and tend to spread out and disperse to look for clean fruits and hosts across pepper fields later in the season. This is a possible reason why pepper weevil distribution is no longer aggregated later in the season.

Pheromone production varies between insects as they are guided by environmental and reproductive age factors. In the case of the tomato leafminer, *Tuta absoluta* Meyrick (Lepidoptera: Gelechiidae), young females produce a higher amount of the major component of their sex pheromone *E*3,*Z*8,*Z*11–14:Ac (TDTA) compared to females older than 11 days [45]. In the pepper weevil, this could affect the egg-laying cycle and the number of eggs oviposited. This variability could also affect the pepper weevil host finding and delay their arrival to a specific part of the field. Lab studies have also confirmed that there is a fluctuation in egg-laying behavior at different times, ages, and with different temperatures (personal observation).

Various adult weevils in the family Curculionidae were reported to follow a negative binomial distribution pattern, which signified a clumped or aggregated distribution. These include the alfalfa weevil, *Hypera postica* [32], the red palm weevil *Rhynchophorus ferrugineus* Oliv. [46], the sunflower seed weevil, *Smicronyx fulvus* LeConte [47], and the pea leaf weevil, *Sitona lineatus* (L.) [48].

This study demonstrated the first step to understanding the spatial distribution of pepper weevil in pepper fields using infested fruit count rather than the usual direct adult count. However, further research is needed to better understand spatial patterns of pepper weevils, especially in larger areas, in fields with different production practices, across all production seasons from the first-time the infestation is obvious until the end of the season, as well as within plant distribution in pepper fields.

## Figures and Tables

**Table 1:** Profile of study field, GPS address, pepper variety, and planting time.

Year	Field	Pepper Type	GPS Location	Planting Time
2021	1	Jalapeño	25.62°, −80.47°	October 2020
2021	2	Long hot	25.62°, −80.47°	October 2020
2021	3	Jalapeño	25.58°, −80.52°	November 2020
2021	4	Long hot	25.58°, −80.52°	November 2020
2022	5	Jalapeño	25.62°, −80.49°	October 2021
2022	6	Long hot	25.62°, −80.49°	October 2021
2023	7	Long hot	25.43°, −80.52°	October 2022

**Table 2:** Statistics and indices to determine distribution pattern.

Indices	Random	Aggregation	Uniform/Regular
Moran’s I	Insignificant *p* value	Significant *p* value and positive Z	Significant *p* value and negative Z
Geary’s C	Values near 1	Values = 0	Values near 2
SADIE analysis	Ia = 1	Ia > 1	Ia < 1

**Table 3:** Comparison of the prediction of Moran’s I and Geary’s C indices for jalapeño and long hot pepper fields.

Field X/Pepper Type	Index	Date 1 (9 WAP)	Date 2 (10 WAP)	Date 3 (11 WAP)	Date 4 (12 WAP)	Date 5 (13 WAP)	Date 6 (14 WAP)
Field 1 Jalapeño	Moran’s I	0.009, Z = 3.23, *p* = 0.0012	0.020, Z = 6.16, *p* < 0.0001	0.018, Z = 4.88, *p* < 0.0001	0.009, Z = 3.12, *p* = 0.0018	0.001, Z = 1.606, *p* = 0.11	
	Geary’s C	1.030, Z = 2.04, *p* = 0.04	0.961, Z = −3.14, *p* = 0.0017	0.972, Z = −2.21, *p* = 0.03	0.9865, Z = −1.07, *p* = 0.28	0.987, Z = −0.996, *p* = 0.32	
Field 2 Long Hot	Moran’s I	0.017, Z = 4.73, *p* < 0.0001	0.013, Z = 3.93, *p* < 0.0001	0.017, Z = 4.8, *p* < 0.0001	0.017, Z = 4.84, *p* < 0.0001		
	Geary’s C	0.878, Z = −9.67, *p* < 0.0001	0.976, Z = −1.88, *p* = 0.0606	0.960, Z = −3.16, *p* = 0.0016	0.968, Z = −2.51, *p* = 0.0120		
Field 3 Jalapeño	Moran’s I	0.0006, Z = 1.52, *p* = 0.13	0.005, Z = 2.41, *p* = 0.02	0.006, Z = 2.59, *p* = 0.0096	0.002, Z = 1.811, *p* = 0.07		
	Geary’s C	0.975, Z = −2.02, *p* = 0.04	0.96, Z = −3.50, *p* = 0.0005	0.980, Z = −1.56, *p* = 0.12	1.000, Z = 0.785, *p* = 0.43		
Field 4 Long Hot	Moran’s I	0.042, Z = 9.83, *p* < 0.0001	0.028, Z = 6.94, *p* < 0.0001	0.011, Z = 3.67, *p* = 0.0002	0.002, Z = 1.72, *p* = 0.08		
	Geary’s C	0.927, Z = −5.79, *p* < 0.0001	0.960, Z = −3.17, *p* = 0.0015	1.000, Z = 0.035, *p* = 0.97	0.977, Z = −1.81, *p* = 0.07		
Field 5 Jalapeño	Moran’s I	−0.003, Z = 0.7, *p* = 0.48	0.003, Z = 2.07, *p* = 0.04	0.060, Z = 13.32, *p* < 0.0001	0.005, Z = 2.36, *p* = 0.02	0.040, Z = 9.61, *p* < 0.0001	−0.002, Z = 0.985, *p* = 0.32
	Geary’s C	0.999, Z = −0.0736, *p* = 0.94	0.980, Z = −1.06, *p* = 0.29	0.890, Z = −8.58, *p* < 0.0001	0.990, Z = −0.978, *p* = 0.33	0.960, Z = −3.40, *p* = 0.0007	0.986, Z = −1.1, *p* = 0.27
Field 6 Long Hot	Moran’s I	0.008, Z = 2.97, *p* = 0.0030	0.037, Z = 8.86, *p* < 0.0001	0.040, Z = 9.39, *p* < 0.0001	0.013, Z = 3.95, *p* < 0.0001	0.020, Z = 4.61, *p* < 0.0001	
	Geary’s C	0.950, Z = −4.02, *p* < 0.0001	0.920, Z = −5.96, *p* < 0.0001	0.933, Z = −5.27, *p* < 0.0001	0.960, Z = −3.53, *p* = 0.0004	0.980, Z = −1.31, *p* = 0.19	
Field 7 Long Hot	Moran’s I	0.016, Z = 10.92, *p* < 0.0001					
	Geary’s C	0.980, Z = −3.26, *p* = 0.0011					

WAP: weeks after planting.

**Table 4:** SADIE index for both jalapeño and pepper fields across various sampling dates.

Field/Year	Pepper Type	Sampling Week	Ia	Pa	Dis Pattern
1/Year 1	Jalapeño	1 (9 WAP)	1.34	0.04	AGG
		2 (10 WAP)	1.39	0.01	AGG
		3 (11 WAP)	1.69	*p* < 2.22 × 10^−16^	AGG
		4 (12 WAP)	0.98	0.49	RAN
		5 (13 WAP)	0.91	0.61	REG
2/Year 1	Long hot	1 (9 WAP)	1.4	0.04	AGG
		2 (10 WAP)	1.3	0.08	AGG
		3 (11 WAP)	0.9	0.44	REG
		4 (12 WAP)	1.07	0.31	RAN
3/Year 1	Jalapeño	1 (9 WAP)	1.39	0.03	AGG
		2 (10 WAP)	1.22	0.18	RAN
		3 (11 WAP)	1.43	0.06	AGG
		4 (12 WAP)	1.28	0.07	RAN
4/Year 1	Long hot	1 (9 WAP)	2.19	*p* < 2.22 × 10^−16^	AGG
		2 (10 WAP)	2.0	*p* < 2.22 × 10^−16^	AGG
		3 (11 WAP)	1.24	0.15	RAN
		4 (12 WAP)	1.05	0.3	RAN
5/Year 2	Jalapeño	1 (9 WAP)	1.36	0.06	AGG
		2 (10 WAP)	0.94	0.57	RAN
		3 (11 WAP)	2.06	*p* < 2.22 × 10^−16^	AGG
		4 (12 WAP)	1.09	0.32	RAN
		5 (13 WAP)	1.65	0.02	AGG
		6 (14 WAP)	1.29	0.08	RAN
6/Year 2	Long hot	1 (9 WAP)	1.4	0.02	AGG
		2 (10 WAP)	1.46	0.02	AGG
		3 (11 WAP)	1.96	*p* < 2.22 × 10^−16^	AGG
		4 (12 WAP)	1.38	0.07	AGG
		5 (13 WAP)	1.59	*p* < 2.22 × 10^−16^	AGG
7/Year 3	Long hot	1 (9 WAP)	2.09	*p* < 2.22 × 10^−16^	AGG

WAP: weeks after planting, dis: distribution, AGG: aggregated, REG: regular, RAN: random.

## Data Availability

Data supporting reported results can be made available upon request from the corresponding authors.

## References

[1] Elmore J.C., Davis A.C., Campbell R.E. (1934). The Pepper Weevil.

[2] Rodriguez-Leyva E. (2006). Life History of *Triaspis eugenii* Wharton and Lopez-Martinez (Hymenoptera: Braconidae) and Evaluation of Its Potential for Biological Control of Pepper Weevil *Anthonomus eugenii* Cano (Coleoptera: Curculionidae). Ph.D. Thesis.

[3] Addesso K.M., McAuslane H.J., Alborn H.T. (2011). Attraction of pepper weevil to volatiles from damaged pepper plants. Entomol. Exp. Appl..

[4] Russell C.J. (2021). Improved Detection and Monitoring of Pepper Weevil (*Anthonomus eugenii*) in Ontario Peppers. Master’s Thesis.

[5] Andrews K.L., Rueda A., Gandini G., Evans S., Arango A., Avedillo M. (1986). A supervised control program for the pepper weevil, *Anthonomus eugenii* Cano, in Honduras, Central America. Int. J. Pest Manag..

[6] Riley D.G., Schuster D.J., Barfield C.S. (1992). Refined action threshold for pepper weevil adults (Coleoptera: Curculionidae) in bell peppers. J. Econ. Entomol..

[7] Ingerson-Mahar J., Eichinger B., Holmstrom K. (2015). How does pepper weevil (Coleoptera: Curculionidae) become an important pepper pest in New Jersey?. J. Integr. Pest Manag..

[8] Segarra-Carmona A.E., Pantoja A. (1988). Sequential sampling plan, yield loss components and economic thresholds for the pepper weevil, *Anthonomus eugenii* Cano (Coleoptera: Curculionidae). J. Agric. Univ. Puerto Rico.

[9] Cartwright B., Teague T.G., Chandler L.D., Edelson J.V., Bentsen G. (1990). An action threshold for management of the pepper weevil (Coleoptera: Curculionidae) on bell peppers. J. Econ. Entomol..

[10] Riley D.G., Schuster D.J., Barfield C.S. (1992). Sampling and dispersion of pepper weevil (Coleoptera: Curculionidae) adults. Environ. Entomol..

[11] Wantuch H., Kuhar T. (2014). Pepper Weevil.

[12] Southwood T.R.E. (1978). Ecological Methods.

[13] Khan R.A., Seal D.R., Zhang S., Liburd O.E., Srinivasan R., Evans E. (2020). Distribution pattern of thrips (Thysanoptera: Thripidae) and tomato chlorotic spot virus in south Florida tomato fields. Environ. Entomol..

[14] Seal D.R., McSorley R., Chalfant R.B. (1992). Seasonal abundance and spatial distribution of wireworms (Coleoptera: Elateridae) in Georgia sweet potato fields. J. Econ. Entomol..

[15] Pieters E.P. (1973). Spatial Distributions and Sampling of Cotton Arthropods with Special Reference to Sequential Sampling. Ph.D. Thesis.

[16] Rózsa L., Reiczigel J., Majoros G. (2000). Quantifying parasites in samples of hosts. J. Parasitol..

[17] Rabinovich J.E. (1980). Introducción a la Ecología de Poblaciones Animales.

[18] Green R.H. (1966). Measurement of non-randomness in spatial distributions. Res. Popul. Ecol..

[19] Winder L., Alexander C., Griffiths G., Holland J., Woolley C., Perry J. (2019). Twenty years and counting with SADIE: Spatial analysis by distance indices software and review of its adoption and use. Rethink. Ecol..

[20] Maruyama Y. (2015). An alternative to Moran’s I for spatial autocorrelation. arXiv.

[21] Peret M., Dittmar P., Agehara S., Smith H. (2021). Vegetable Production Handbook of Florida, 2021–2022.

[22] Getis A. (2007). Reflections on spatial autocorrelation. Reg. Sci. Urban Econ..

[23] Aswi A., Cramb S., Duncan E., Mengersen K. (2021). Detecting spatial autocorrelation for a small number of areas: A practical example. J. Phys..

[24] Mathur M. (2015). Spatial autocorrelation analysis in plant population: An overview. J. Appl. Nat. Sci..

[25] Wong D.W.S., Lee J. (2005). Statistical Analysis of Geographic Information with ArcView GIS and ArcGIS.

[26] Zhang D., Mao X., Meng L. (2010). A method using ESDA to analyze the spatial distribution patterns of cultural resource. Int. Arch. Photogramm. Remote Sens. Spat. Inf. Sci..

[27] Cliff A., Ord J.K. (1981). Spatial Processes: Models and Applications.

[28] Perry J.N., Bell E.D., Smith R.H., Woiwod I.P. (1996). SADIE: Software to measure and model spatial pattern. Asp. Appl. Biol..

[29] Perry J.N. (1998). Measures of spatial pattern for counts. Ecology.

[30] Park Y., Tollefson J.J. (2005). Characterization of the spatial dispersion of corn root injury by corn rootworms (Coleoptera: Chrysomelidae). J. Econ. Entomol..

[31] Martins J.C., Picanco M.C., Silva R.S., Gonring A.H.R., Galdino T.V.S., Guedes R.N.C. (2017). Assessing the spatial distribution of *Tuta absoluta* (Lepidoptera: Gelechiidae) eggs in open-field tomato cultivation through geostatistical analysis. Pest Manag. Sci..

[32] Shrestha G., Rijal J.P., Reddy G.V.P. (2020). Characterization of the spatial distribution of alfalfa weevil, *Hypera postica*, and its natural enemies, using geospatial models. Pest Manag. Sci..

[33] Rijal J.P., Brewster C.C., Bergh J.C. (2014). Spatial distribution of grape root borer (Lepidoptera: Sesiidae) infestations in Virginia vineyards and implications for sampling. Environ. Entomol..

[34] Perry J.N. (1995). Spatial analysis by distance indices. J. Anim. Ecol..

[35] Perry J.N. (1997). Spatial association for counts of two species. Acta Jutl..

[36] Wickham H., Hester J., Chang W., Bryan J. (2022). Devtools-package: Devtools: Tools to Make Developing R Packages Easier. R Package Version 2.4.5. https://CRAN.R-project.org/package=devtools.

[37] Gigot C. (2023). Analyzing Plant Disease Epidemics with the R Package Epiphy. R Package Version 0.5.0.9000. https://CRAN.R-project.org/package=epiphy.

[38] SAS Institute (2013). SAS/STAT 9.4 User’s Guide.

[39] Eller F.J., Bartelt R.J., Shasha B.S., Schuster D.J., Riley D.G., Stansly P.A., Mueller T.F., Shuler K.D., Davis J.H., Sutherland C.A. (1994). Aggregation pheromone for the pepper weevil, *Anthonomus eugenii* Cano (Coleoptera: Curculionidae): Identification and field activity. J. Chem. Ecol..

[40] Eller F.J., Palmquist D.E. (2014). Factors Affecting Pheromone Production by the Pepper Weevil, *Anthonomus eugenii* Cano (Coleoptera: Curculionidae) and collection efficiency. Insects.

[41] Bandeira P.T., Favaro C.F., Francke W., Bergmann J., Zarbin P.H.G. (2021). Aggregation pheromones of weevils (Coleoptera: Curculionidae): Advances in the identification and potential uses in semiochemical-based pest management strategies. J. Chem. Ecol..

[42] Addesso K.M., McAuslane H.J. (2009). Pepper weevil attraction to volatiles from host and nonhost plants. Environ. Entomol..

[43] Giblin-Davis R.M., Oehlschlager A.C., Perez A., Gries G., Gries R., Weissling T.J., Chinchilla C.M., Peña J.E., Hallett R.H., Pierce H.D. (1996). Chemical and behavioral ecology of palm weevils (Curculionidae: Rhynchophorinae). Fla. Entomol..

[44] Addesso K.M., Alborn H.T., Bruton R.R., McAuslane H.J. (2021). A multicomponent marking pheromone produced by the pepper weevil, *Anthonomus eugenii* (Coleoptera: Curculionidae). Chemoecology.

[45] Dominguez A., Lopez S., Bernabe A., Guerrero A., Quero C. (2019). Influence of age, host plant and mating status in pheromone production and new insights on perception plasticity in *Tuta absoluta*. Insects.

[46] Faleiro J.R., Kumar J.A., Rangnekar P.A. (2002). Spatial distribution of red palm weevil *Rhynchophorus ferrugineus* Oliv. (Coleoptera: Curculionidae) in coconut plantations. Crop Prot..

[47] Peng C., Brewer G.J. (1994). Distribution of the red sunflower seed weevil (Coleoptera: Curculionidae) on sunflower. Environ. Entomol..

[48] Schotzko D.J., Quisenberry S.S. (1999). Pea leaf weevil (Coleoptera: Curculionidae) spatial distribution in peas. Environ. Entomol..

